# Benchmarking Low-Frequency Variant Calling With Long-Read Data on Mitochondrial DNA

**DOI:** 10.3389/fgene.2022.887644

**Published:** 2022-05-19

**Authors:** Theresa Lüth, Susen Schaake, Anne Grünewald, Patrick May, Joanne Trinh, Hansi Weissensteiner

**Affiliations:** ^1^ Institute of Neurogenetics, University of Lübeck and University Hospital Schleswig-Holstein, Lübeck, Germany; ^2^ Luxembourg Centre for Systems Biomedicine, University of Luxembourg, Belvaux, Luxembourg; ^3^ Institute of Genetic Epidemiology, Medical University of Innsbruck, Innsbruck, Austria

**Keywords:** nanopore sequencing, long-read, mtDNA, heteroplasmy, benchmarking, mixtures, haplogroups, low-frequency variant

## Abstract

**Background:** Sequencing quality has improved over the last decade for long-reads, allowing for more accurate detection of somatic low-frequency variants. In this study, we used mixtures of mitochondrial samples with different haplogroups (i.e., a specific set of mitochondrial variants) to investigate the applicability of nanopore sequencing for low-frequency single nucleotide variant detection.

**Methods:** We investigated the impact of base-calling, alignment/mapping, quality control steps, and variant calling by comparing the results to a previously derived short-read gold standard generated on the Illumina NextSeq. For nanopore sequencing, six mixtures of four different haplotypes were prepared, allowing us to reliably check for expected variants at the predefined 5%, 2%, and 1% mixture levels. We used two different versions of Guppy for base-calling, two aligners (i.e., Minimap2 and Ngmlr), and three variant callers (i.e., Mutserve2, Freebayes, and Nanopanel2) to compare low-frequency variants. We used F_1_ score measurements to assess the performance of variant calling.

**Results:** We observed a mean read length of 11 kb and a mean overall read quality of 15. Ngmlr showed not only higher F_1_ scores but also higher allele frequencies (AF) of false-positive calls across the mixtures (mean F_1_ score = 0.83; false-positive allele frequencies < 0.17) compared to Minimap2 (mean F_1_ score = 0.82; false-positive AF < 0.06). Mutserve2 had the highest F_1_ scores (5% level: F_1_ score >0.99, 2% level: F_1_ score >0.54, and 1% level: F_1_ score >0.70) across all callers and mixture levels.

**Conclusion:** We here present the benchmarking for low-frequency variant calling with nanopore sequencing by identifying current limitations.

## 1 Introduction

While next-generation sequencing (NGS) allowed cost-effective sequencing of whole-genome sequences over the last decades, third-generation sequencing (TGS) has finally made it possible to generate the first complete assembly of a human genome (Nurk et al., 2022). The improvement in TGS resulted mainly from improved single-molecule long-read sequencing chemistry and newly developed bioinformatic methods enabling the analysis of reads at >10 kb (Logsdon et al., 2020). We investigate whether Oxford Nanopore Technologies(ONT) achieves the base-calling accuracy needed for low-frequency variant calling. A plethora of tools for somatic (i.e., low-frequency) variant calling exist for NGS data, thereby allowing detection of low-frequency variants in short-read data even below the 1% variant allele frequency (Schmitt et al., 2012; Kennedy et al., 2013). However, due to the difference in applications, no variant caller stands out for all the different scenarios (Koboldt, 2020).

Numerous available tools for TGS are tracked on long-read-tools.org (Amarasinghe et al., 2021), currently listing 80 aligners, 101 *de-novo* assemblers, and 15 tools useful for both approaches. For base-calling, 36 different tools are available for Oxford Nanopore Technology (ONT), while for SNP and variant analysis, 109 tools/pipelines are currently available. In contrast to the high number of available tools, only six comparative studies are presently available that benchmark long-read data analysis tools/platforms (Wick and Holt, 2019; Latorre-Perez et al., 2020; Møller et al., 2020; Liu et al., 2021; Pei et al., 2021; Yuen et al., 2021). In fact, only two studies focus on benchmarking variant callers for long-read sequencing data. While Møller *et al.* focused on diploid variant callers (Medaka, Clair, Pepper/Deepvariant) (https://github.com/nanoporetech/medaka; Poplin et al., 2018; Luo et al., 2020; Shafin et al., 2021), Pei *et al.* benchmarked both next-generation sequencing (NGS) and TGS data with 11 different variant callers in mixtures down to 10%, however limiting the TGS tools for germline variant calling with DNAseq and GATKs HaplotypeCaller. Thereby for the somatic haplotype calling, only NGS data was used for benchmarking purposes.

Thus far, benchmarking for long-read sequencing data and low-frequency variant callers has not been performed below the 5% mixtures. When reviewing the present literature, there are 15 pre-prints, 20 research articles, one book chapter, and two protocols related to mtDNA and nanopore long-read sequencing. When focussing on human mtDNA, Lindberg et al., 2016 investigated mtDNA data on a MinION device, with mixtures at the 1:1 ratio (Lindberg et al., 2016), and Zascavage *et al.* present protocols for mtDNA analysis on ONT devices and results with error rates of 0.3% per mtDNA sequence–corresponding to 50 base-substitution errors per sample (Zascavage RR. et al., 2019; Zascavage R. R. et al., 2019). Nakanishi *et al.* recently analyzed mtDNA haplotypes in mixed DNA samples on MinION and MiSeq in a manual fashion, down to 5% (Nakanishi et al., 2022). Furthermore, mtDNA methylation has been investigated by Bicci *et al.* (Bicci et al., 2021) and Lüth *et al.* (Luth et al., 2021).

Mitochondrial DNA has a ∼10x higher mutation rate than nuclear DNA (Pinto and Moraes, 2015). Its content per cell ranges from 100s to several 1,000 molecules depending on the tissue (Wachsmuth et al., 2016). The circular 16.6 kb mtDNA is maternally inherited and is recombination-free (Wei et al., 2020). These features render it an ideal model for benchmarking low-frequency variant calling since we can mix two haplotypes at any ratio. Thereby mitochondrial homoplasmic variants, which are variants present in all mtDNA copies, define an individual’s haplotype. Rare homoplasmic variants are described as causing diseases like LHON, MELAS (Goto et al., 1990; Bargiela and Chinnery, 2019). The accumulation of mitochondrial heteroplasmic variants (i.e. variants that are present in a fraction of the mtDNA copies) is associated with aging as well as various diseases such as neurological and cardiovascular, cancer and diabetes (Stewart and Chinnery, 2021). At the population level, Laricchia *et al.* analyzed 56,434 mtDNA samples, where most samples showed no heteroplasmy; however, one in 250 samples carried pathogenic heteroplasmic variants above the 10% (Laricchia et al., 2022). Bolze *et al.* analyzed ∼200,000 mitochondrial genomes between 5% and 1% and identified one heteroplasmic variant per person on average (range from 0 to 13, median 0) (Bolze et al., 2020).

In this study, we deeply investigate low-frequency variants calling for long-read data, as it is of great importance to investigate heteroplasmy and somatic variants accurately. First, we prepared a gold standard from NGS data of mitochondrial genomes by merging the results from three different variant callers. Subsequently, the TGS mixtures were prepared, base-called, trimmed, and additional quality control (QC) was performed. Finally, the performance of two variant callers designed for short-read data and one low-level variant specific for long-read data was compared. We evaluated the performance of the callers to detect low-frequency variants in mtDNA mixtures at predefined 5%, 2%, and 1% levels. In different short-read sequencing-based studies, the sensitivity thresholds to detect heteroplasmy ranged from >10% to 0.1% (Li et al., 2010; Guo et al., 2013; Dierckxsens et al., 2020; Fazzini et al., 2021; Weissensteiner et al., 2021). In addition, short-read duplex sequencing enabled the reliable detection of heteroplasmic variants at 0.01% (Ahn et al., 2015). However, as we are aware of the lower base-calling quality of nanopore sequencing, we did not include mixtures below the 1% minor component percentage. We also highlight the advantages of employing different mtDNA haplotypes as validation sets, with mean read lengths at 11 kb.

## 2 Methods

### 2.1 Library Preparation and Sequencing

#### 2.1.1 DNA Extraction and Sample Selection

**FIGURE 1:**
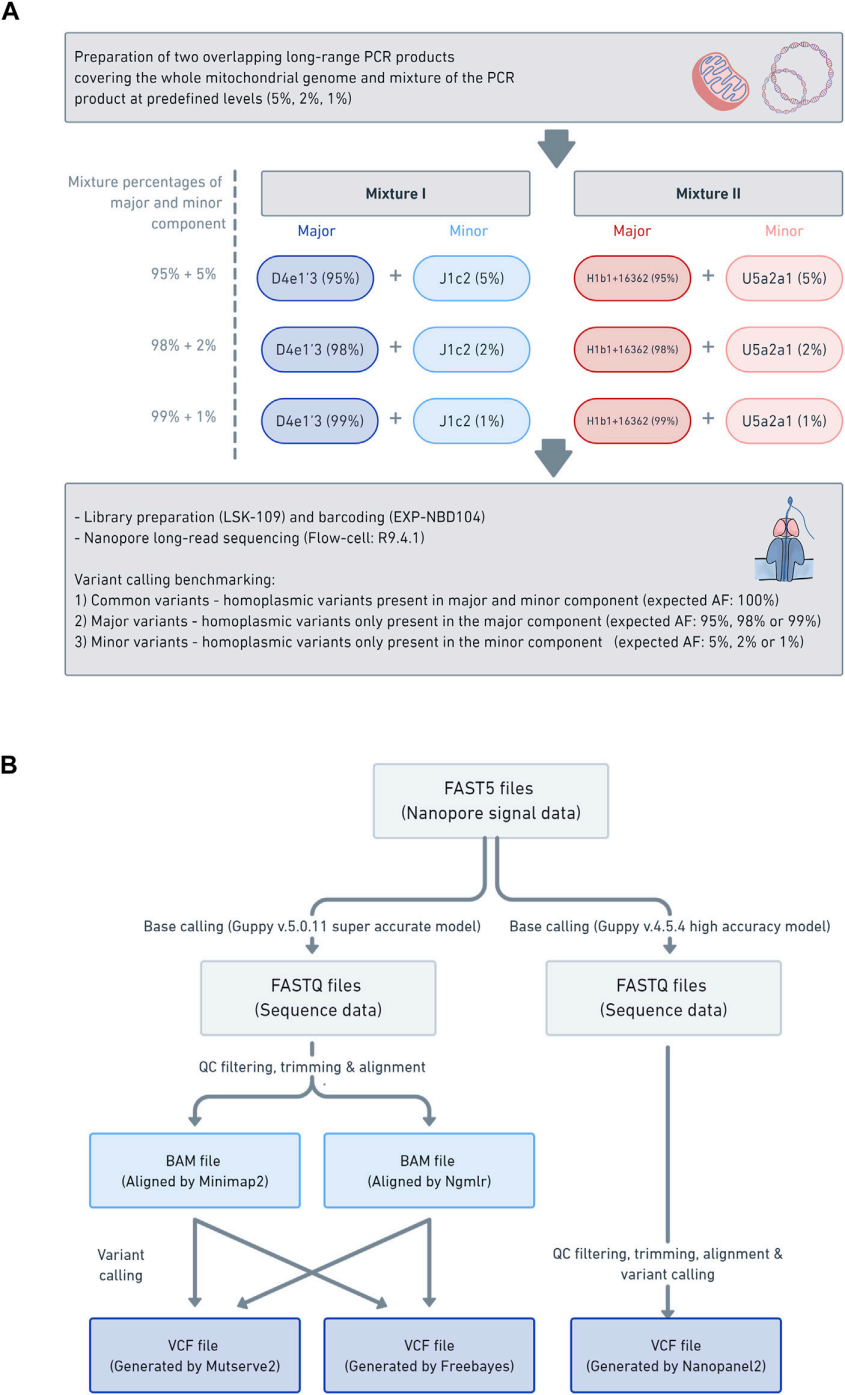
Laboratory workflow data analysis pipeline **(A)** The schema illustrates the laboratory workflow for the preparation of the mitochondrial DNA (mtDNA) mixture models and subsequent nanopore sequencing **(B)** Workflow of how the mtDNA nanopore data was processed and which aligners (i.e., Minimap2 and Ngmlr) and variant callers (i.e., Mutserve2, Freebayes, and Nanopanel2) were used.

All DNA was extracted from blood with the Blood and Cell Culture DNA Midi kit (Qiagen). Four samples (B-28, L-2804, L-3034, and L-649) were used in this study for the mixture models. The selected samples were from participants of European descent (Supplementary Table S1). We have combined haplogroups (D4e1′3 and J1c2) and (H1b1+16362 and U5a2a1) based on the phylogenetic distance (16 branches apart for each mixture) and the number of obtained minor variants (>18). See Supplementary Figure S1 for additional information on SNPs (shared, major, and minor variants annotated according to coding-, control-region, rRNA, tRNA) and intermediate haplogroups, as well as the gold standard in Supplementary Table S2. Two different mixtures with different percentages were prepared at 1%, 2%, and 5% based on concentration (Table 1; Figure 1A).

**TABLE 1:** Summary of detected minor variants, false-positive calls, and F1 scores resulting from the three tested variants callers.

Mixture and haplogroup	Mixture I: D4e1′3 and J1c2			Mixture II: H1b1+16362 and U5a2a1		
Percentage major and minor component	95% + 5%	98% + 2%	99% + 1%	95% + 5%	98% + 2%	99% + 1%
**Mutserve2 (alignment generated with Minimap2)**						
** Detected minor variants/expected minor variants**	18/18	15/18	11/18	21/22	1/22	14/22
** False-positive variant calls**	2	4	13	1	3	12
** F**_**1**_ **score**	0.98	0.93	0.82	0.97	0.52	0.72
**Mutserve2 (alignment generated with ngmlr)**						
** Detected minor variants/expected minor variants**	18/18	17/18	11/18	21/22	2/22	13/22
** False-positive variant calls**	1	2	14	2	3	12
** F**_**1**_ **score**	0.99	0.96	0.81	0.99	0.54	0.70
**Freebayes (alignment generated with Minimap2)**						
** Detected minor variants/expected minor variants**	18/18	18/18	15/18	22/22	2/22	18/22
** False-positive variant calls**	11	21	83	8	21	99
** F**_**1**_ **score**	0.89	0.83	0.34	0.89	0.41	0.37
**Freebayes (alignment generated with ngmlr)**						
** Detected minor variants/expected minor variants**	18/18	18/18	12/18	22/22	3/22	20/22
** False-positive variant calls**	28	73	337	20	56	265
** F**_**1**_ **score**	0.79	0.59	0.21	0.77	0.29	0.19
**Nanopanel2 (alignment generated with Minimap2)**						
** Detected minor variants/expected minor variants**	14/18	13/18	3/18	16/22	1/22	8/22
** False-positive variant calls**	2	2	19	1	2	22
** F**_**1**_ **score**	0.86	0.85	0.61	0.77	0.33	0.43
**Nanopanel2 (alignment generated with ngmlr)**						
** Detected minor variants/expected minor variants**	14/18	12/18	4/18	15/22	1/22	8/22
** False-positive variant calls**	3	2	14	1	1	12
** F**_**1**_ **score**	0.85	0.84	0.64	0.75	0.33	0.47

Variant callers: Mutserve2, Freebayes and Nanopanel2, Aligners: Minimap2 and Ngmlr.

Bold values are headlines for better discrimination between mixtures, aligners and variant callers.

#### 2.1.2 Illumina NextSeq Short-Read Sequencing

MtDNA was amplified by two overlapping long-range PCRs, which were subsequently tagmented and sequenced on an Illumina NextSeq. Deep mitochondrial sequencing was performed with bait enrichment or long-range PCR, two primer sets used for the long-range PCR were: MTL-F1 5′- AAA GCA CAT ACC AAG GCC AC -3′, MTL-F2 5′- TAT CCG CCA TCC CAT ACA TT -3′, MTL-R1 5′- TTG GCT CTC CTT GCA AAG TT -3′, MTL-R2 5′- AAT GTT GAG CCG TAG ATG CC -3’. Next-generation sequencing was performed on the NextSeq500 (Illumina, Inc.) to produce 2 × 150 bp reads. Raw sequencing reads were converted to standard FASTQ format using bcl2fastq software 2.17.1.14 (Illumina, Inc.). Raw FASTQ files were analyzed with FastQC and aligned with BWA MEM to the mitochondrial reference genome (rCRS). The resulting SAM files were processed with SAMtools to sorted BAM files. Subsequently, the BAM files were processed with GATK, and duplicates were removed with MarkDuplicates. Additional quality control was performed with QualiMap2 and afterward, MultiQC was applied to the resulting reports, including the FastQC reports.

#### 2.1.3 Oxford Nanopore Long-Read Sequencing

Two overlapping amplicons spanning the complete mtDNA were prepared with long-range PCRs, using the same primer sets for Illumina sequencing. Please see Supplementary Table S3, S4 for specific PCR mix and conditions. The DNA concentration was measured and subsequently normalized. Next, the PCR products of the major component (PCR product of sample: B-28 and L-3034, haplogroup: D4e1′3 or H1b1+16362) were mixed with the minor component (PCR product of sample: L-2804 and L-649, haplogroup: J1c2 or U5a2a1) at the predefined levels of 5%, 2% and 1% (Supplementary Table S5). Subsequently, each mixture was barcoded with the Native 1–12 Barcoding Kit (EXP-NBD104) and multiplexed, using 400 ng of each mixture. The library was prepared with the Ligation Sequencing Kit (SQK-LSK109), following the manufacturer’s instructions. The library of the six barcoded mixture samples was loaded on one R9.4.1 flow cell and sequenced on the GridION. Base-calling was performed with Guppy v5.0.11 with the super-accurate model and v4.5.4 with the high accuracy model. The base-called reads were filtered with Filtlong (v0.2.0) (https://github.com/rrwick/Filtlong) to only include the best 50% of the reads, based on Phred quality scores (q-score), with a minimum read length of 9 kb. Then, the reads were trimmed with NanoFilt (v2.5.0) (De Coster et al., 2018) and 75bp were cropped from the front of the reads and 20bp from the end. Subsequently, the nanopore reads were aligned against the mitochondrial genome reference sequence (rCRS) using Minimap2 (v2.17) (Li, 2021) and Ngmlr (v0.2.7) (Sedlazeck et al., 2018). Finally, the alignments were sorted and indexed with Samtools (v1.3.1) (Danecek et al., 2021).

### 2.2 Data Analysis

#### 2.2.1 Preparation of Gold Standard From NGS

The processed BAM files previously aligned against the rCRS were analyzed with three different variant callers: GATK v4 Mutect2 (Benjamin et al., 2019), Freebayes v.1.3.4 (arXiv:1207.3907), and Mutserve2 c.2.0.0-rc.12 (Weissensteiner et al., 2021). We applied an alignment score of Q30 and set the variant level at 0.7% (where applicable). For GATK Mutect2, the mitochondria flag was applied. The resulting VCF files were processed with BCFtools *query* and subsequently merged into consensus variants (Li, 2011). The four individuals (B-28, L-2804, L-3034, and L-649) had haplogroups D4e1′3, J1c2, H1b1+16362, and U5a2a1, respectively, estimated with HaploGrep2 (Weissensteiner et al., 2016b). Please see Supplementary text 1 for more details on the exact commands.

We distinguished between three different variant types, requiring a variant to be detected with at least two different variant callers: 1) Common Variants are homoplasmic variants found in both samples from a mixture.2) Major Variants are all variants found in the major contribution of a mixture, expected to be present at 99%, 98%, and 95% in the 1%, 2%, and 5% mixtures, respectively, further including private mutations at lower levels. 3) Minor Variants are all variants found in the minor contribution of the mixture, expected to be present at 1%, 2%, and 5%. Supplementary Table S2 lists the expected variants (gold standard) for the mixtures.

#### 2.2.2 Comparison of Mixtures and Callers

The performance of two variant callers designed for short-read data (i.e. Mutserve2 and Freebayes) and one low-level variant specific for long-read data (Nanopanel2) was compared. We chose Mutserve2 (Weissensteiner et al., 2016a), as the tool has performed well in previous work (Fazzini et al., 2021; Ip et al., 2022) and only NOVOPlasty, exclusively designed for short-read data, outperformed Mutserve2 (Dierckxsens et al., 2020). Freebayes (arXiv:1207.3907v2 [q-bio.GN]) was selected because we were interested in a haplotype-aware variant caller that is also designed for somatic variants and Freebayes was previously used for both long-read and short-read data (Ebler et al., 2019). Lastly, we screened the literature for long-read specific low-frequency variant callers and Nanopanel2 (Popitsch et al., 2021) was the only one applicable for our study at the time.

#### 2.2.3 Variant Calling From Nanopore Long-Read Sequencing

In order to assess the applicability of nanopore long-read sequencing for low-frequency variant calling, three software tools, chosen based on various strengths and weaknesses, were compared (i.e., Mutserve2, Freebayes, and Nanopanel2) (Figure 1B). The alignments in a BAM format, prepared with either Minimap2 or Ngmlr, were individually processed with Mutserve2 (v2.0.0) (Weissensteiner et al., 2016a) and Freebayes (v1.3.4) (arXiv:1207.3907v2 [q-bio.GN]). To call variants with Nanopanel2 (v1.01) (Popitsch et al., 2021), the Nanopore FAST5 had to be rebase-called with Guppy v4.5.4 in high accuracy mode. The rebase-called reads were filtered and trimmed as described above and subsequently processed with Nanopanel2. The exact commands for quality filtering, alignment and variant calling are listed in Supplementary Text 1.

#### 2.2.4 Statistical Analysis

To measure the variant calling performance, we stratified the detected variants from the nanopore data as false-positive, true-positive, and false-negative calls. True-positive variants, thus, variants that we expected to be present in the mixtures, were determined with the previously generated Illumina short-read gold standard. On the other hand, false-positive variants are variants that were not identified in the NGS gold standard. False-negative variants were expected to be present in the mixtures but were not detected. Then the F_1_ score was calculated (F_1_ score = 2 × (sensitivity × precision) ÷ (sensitivity + precision)). For calculating the F_1_ score and the determination of false-positive calls, we used the following minimum variant level to filter the output of Mutserve2, Freebayes, and Nanopanel2: ≥0.025 for the 5% mixture, ≥0.015 for the 2% mixture, and ≥0.007 for the 1% mixtures. In addition, to assess the variant calling performance, the number of false positives (n_FP_) was calculated.

## 3 Results

The NGS gold standard was derived as previously formulated (Cortes-Figueiredo et al., 2021; Fazzini et al., 2021) by sequencing on Illumina NextSeq. We tested two mtDNA mixtures (mixture I: D4e1′3 & J1c2, mixture II: H1b1+16362 & U5a2a1) at three predefined levels (5%, 2% and 1%) (Figure 1). We compared the performance of two commonly used aligners (i.e., Minimap2 and Ngmlr) (Ren and Chaisson, 2021) and subsequent variant calling with three tools to detect low-frequency SNVs. For our study, we chose two somatic variant callers originally developed for short-read sequencing data (i.e., Mutserve2, Freebayes) and one tool developed for nanopore long-read sequencing (i.e., Nanopanel2) (Popitsch et al., 2021).

After deep-nanopore sequencing of the mtDNA long-range PCR products, we obtained a mean read q-score of 13.7 and a mean read length of 8.6 kb (SD = ±247.6 bp) across all raw sequencing data, base-called with the Guppy super-accurate model (Supplementary Figure S2A). After the quality and length filtering and read trimming, we obtained a mean read q-score of 14.9 and a mean read length of 11.3 kb (SD = ±350.4 bp) (Supplementary Figure S2B). Next, we separated the sequencing data by base-calling q-score, and with a q-score >20, we obtained coverage of 2937X (±1732X) (Supplementary Figures S3-S5). Rebase-calling with an older Guppy version (v4.5.4) was required to process the variant caller Nanopanel2 and we obtained a mean read q-score of 14.2 and a mean read length of 11.3 kb (SD = ±307.2 bp) with the high accuracy base-calling model.

### 3.1 Comparison of Aligners

For the comparison, we focused on the F_1_ score, the number of false-positive calls (n_FP_), and the number of detected minor (i.e. low-frequency) variants. The common, major, and minor variants expected in the mixture were determined using a previously derived Illumina short-read gold standard. Besides the variant caller, the utilized aligner affected the outcome. First, Ngmlr showed a better performance when used in combination with Mutserve2 with a mean F_1_ score of 0.83 and a total of 34 n_FP_ across all mixtures and levels (Table 1). Minimap2 showed a slightly lower F_1_ score and a higher n_FP_ with Mutserve2 (mean F_1_ score = 0.82, n_FP_ = 35). Second, when variants were called with Freebayes, Minimap2 (mean F_1_ score = 0.81, n_FP_ = 243) performed better compared to Ngmlr (mean F_1_ score = 0.47, n_FP_ = 779). Lastly, for the variant detection with Nanopanel2, Ngmlr (mean F_1_ score = 0.65, n_FP_ = 33) showed the slightly better performance compared to Minimap2 (mean F_1_ score = 0.64, n_FP_ = 48).

### 3.2 Comparison of Variant Callers

**FIGURE 2:**
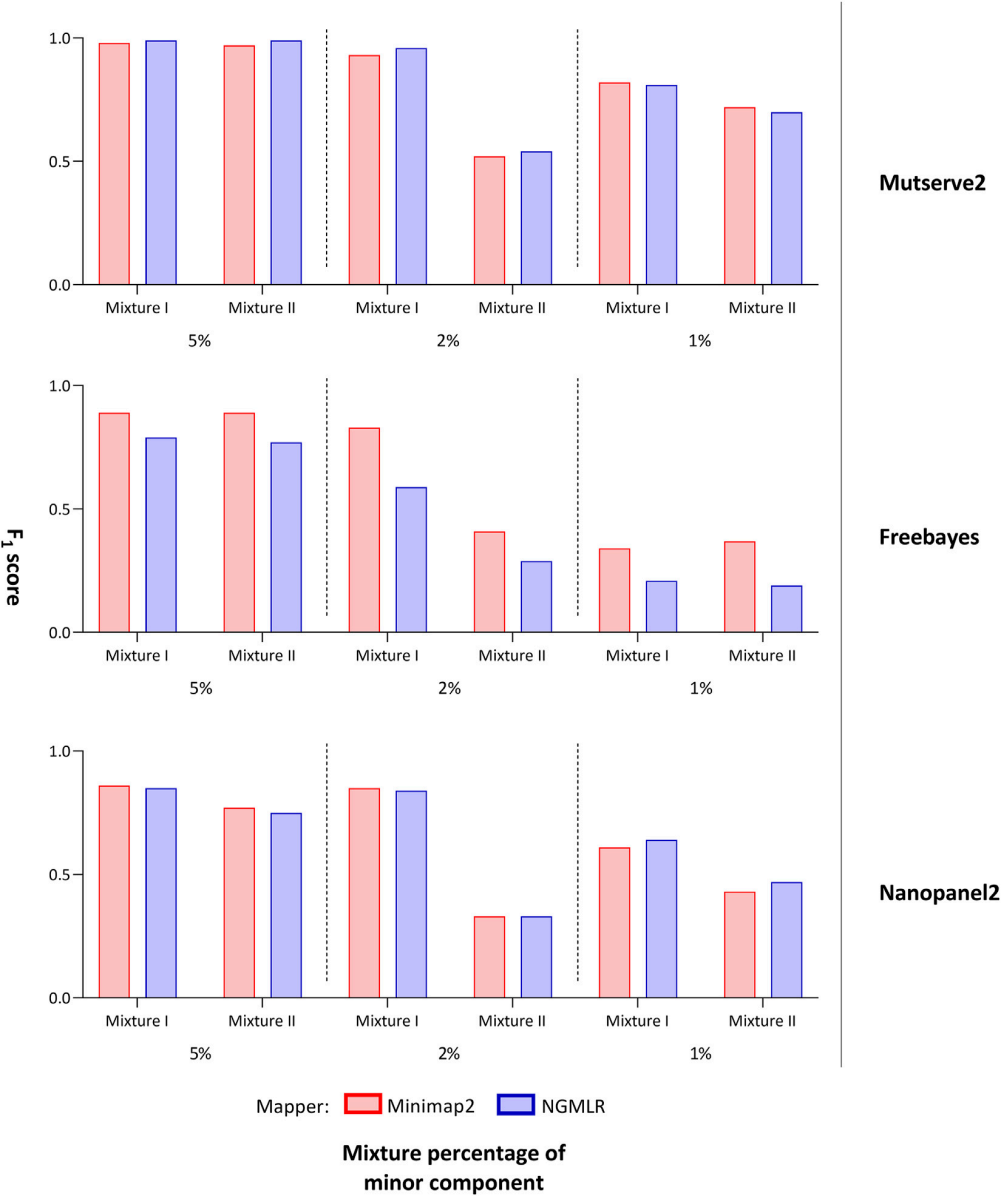
F_1_ score of tested variant callers. Bar chart that shows the F_1_ score of the mitochondrial variants detected with two aligners (i.e., Minimap2 and Ngmlr) and the three variant callers (i.e., Mutserve2, Freebayes, and Nanopanel2).

**FIGURE 3:**
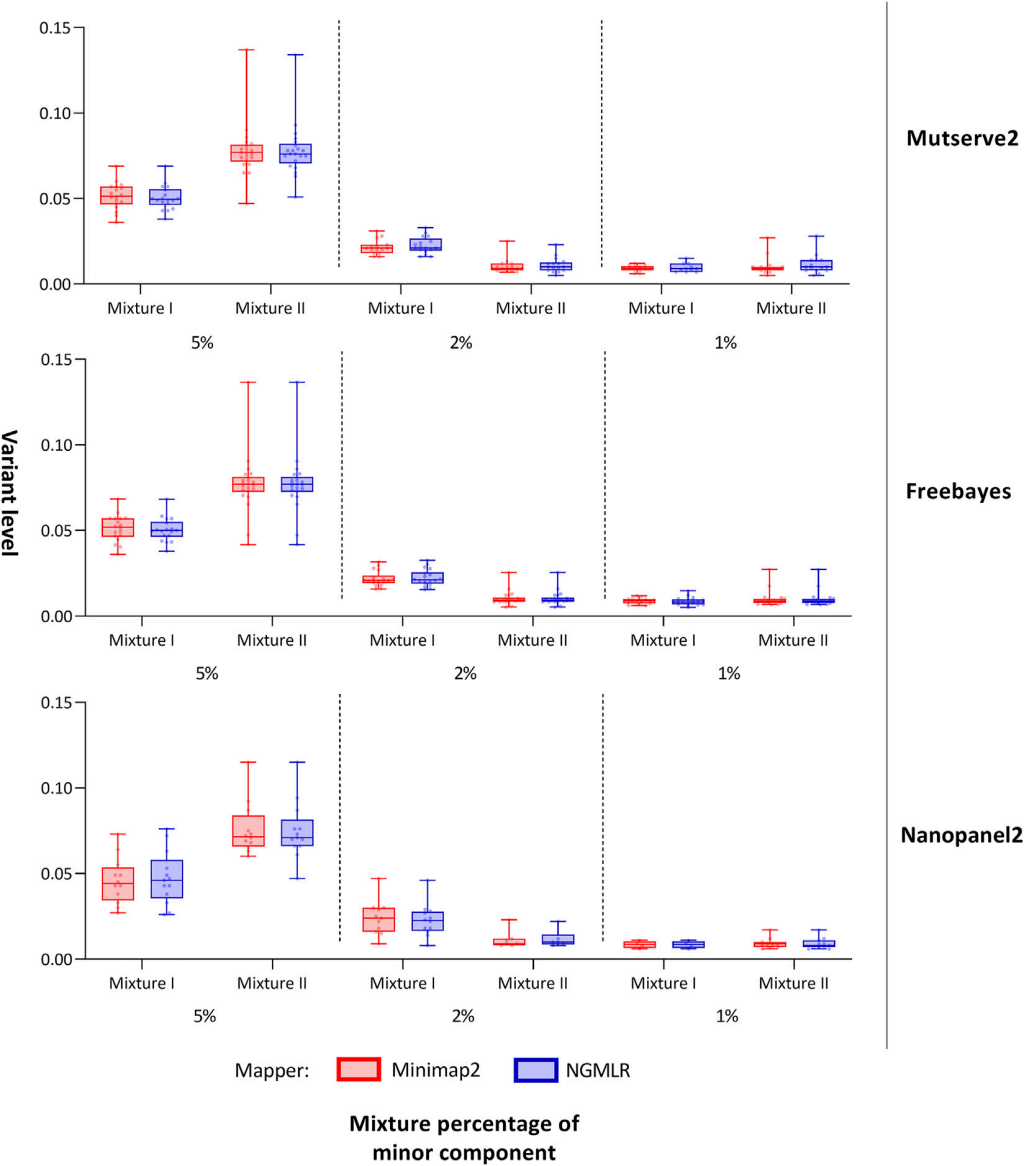
Allele frequency of variants detected from the mitochondrial DNA nanopore data. Box plots show the allele frequency of the mitochondrial minor variants detected with two aligners (i.e., Minimap2 and Ngmlr) and the three variant callers (i.e., Mutserve2, Freebayes, and Nanopanel2). The bars and whiskers represent the median, the interquartile range, and the minimum and maximum.

In our first benchmarking analysis, Mutserve2 in combination with the Ngmlr aligner, showed the best performance to detect low-frequency variants in the mtDNA mixture models. For mixture I, we obtained higher F_1_ scores but the same n_FP_ (mean F_1_ score = 0.92, n_FP_ = 17) compared to mixture II (mean F_1_ score = 0.74, n_FP_ = 17) (Figure 2). Mutserve2 in combination with Ngmlr had the highest F_1_ scores (5%-level: F_1_ score = 0.99, 2%-level: F_1_ score >0.54, 1%-level: F_1_ score >0.70) and lowest n_FP_ (5%-level: n_FP_ < 2, 2%-level: n_FP_ < 3, 1%-level: n_FP_ < 14) across all callers and mixture levels. The lower F_1_ scores in mixture II was mainly driven by the overall lower AF of the minor variants of the 2% mixture (Figure 3). The detected variant AF of the minor variants in mixture I matched the expected levels (5% level: median AF = 0.05, 2% level: median AF = 0.02, 1% level: median AF = 0.01). By contrast, the detected AF from mixture II differed more from the expected corresponding mixture levels (5% level: median AF = 0.08, 2% level: median AF = 0.01, 1% level: median AF = 0.01). The overall detected AF of the minor variants was comparable in all tested variant callers independent of the used aligners. As expected, the lower the minimum AF, the lower the resulting F_1_ score and the higher the n_FP_ (Supplementary Figure S6).

Using Freebayes, we detected significantly higher n_FP_ and therefore, lower F_1_ scores. However, we obtained higher F_1_ scores and lower n_FP_ in the mixture I (mean F_1_ score = 0.69, n_FP_ = 115) compared to mixture II (mean F_1_ score = 0.56, n_FP_ = 128) (Figure 2).

Lastly, Nanopanel2 requires nanopore data from an older Guppy version. Nanopanel2 also showed lower F_1_ scores compared to Mutserve2, which was due to the low number of true-positive calls. Compared to Mutserve2 and Freebayes, Nanopanel2 did not detect all common and major variants. Nanopanel2 performed better with Ngmlr and detected lower n_FP_ compared to Freebayes. On the other hand, the number of true-positive variant calls detected with Nanopanel2 was significantly lower compared to the other two callers (Figure 2) as well. Subsequently, we obtained overall lower F_1_ scores with Nanopanel2 (mixture I: mean F_1_ score = 0.77, n_FP_ = 19; mixture II: mean F_1_ score = 0.51, n_FP_ = 14).

### 3.3 Investigation of False-Positive Variants (Type I Errors)

After removing all expected variants from the results, we further analyzed Type I errors in more detail. Here, we focused on the Q20 filtered data for both mixtures, and both mappers were analyzed with Mutserve2 only. We removed variants around 3107 and 302–316 due to reference issues. A total of 189 false-positive (FP) variants could be observed in the 12 samples (95 FPs with Minimap2 and 94 FPs with Ngmlr) greater or equal to the 0.7% level. Ngmlr yielded the highest false-positive variant levels at 17.4% on position 70 (mean overall variant level 1.69%), whereas Minimap2 showed a 6.2% false-positive variant on position 6991 (mean overall variant level 1.63%). Supplementary Table S6 lists all false-positive variants, including the annotation provided with Mutserve2. Variants on 5747G and 2129A were found most often in the mixtures with both mappers at mean variant levels 0.97% and 2.41%, respectively. Both variants are in low-complexity regions (i.e., polymorphic A-stretches). In total, 46.56% of all false-positive variants are in low-complexity regions (LCR) of at least length 6. Lowering this length threshold to 4, the percentage of false-positives variants in LCR increases to 57.67%. One of such variants is the previously mentioned variant found with Ngmlr on position 70 (in a G-stretch). Overall, the two mixtures share 28.4% of all uniquely found false-positive variants, represented in 100/189 false-positive variants, further underlying that the errors include systematic artifacts. Supplementary Tables S7, S8 show a detailed presentation of false-positive variants per mixture I and II, respectively. The lower coverage in fragment one manifests a reduced number of type I errors (8.4%), with fragment two showing 92 (48.68%) and the overlapping regions 81 (42.85%) false-positive variants (Supplementary Tables 7 and 8).

### 3.4 Investigation of False-Negative Variants (Type II Errors)

For both mixtures, I and II, the expected numbers of variants are 55 and 43, respectively. Applying a different threshold per predefined mixture level, the cut-off levels were set at 0.7%, 1.5%, and 2.5% to interpret the 1%, 2%, and 5% mixtures, respectively, with Mutserve2 (Supplementary Figures S7, 8). We observed a different variant count between false-negatives in fragments one and 2 with 78 and 24 missing variants, respectively (50 variants are missing in overlapping regions, Supplementary Table S9). This difference can be attributed to the unbalanced coverage, with more variants missing in the lower-covered fragment. Although the coverage was higher in mixture II, the amount of false negatives is more pronounced here, mainly due to shifted mixture ratios, lower than originally planned. Lowering the detection cut-off to 0.5% for both the 1% and 2% mixtures in mixtures II (Figure 3 and Supplementary Table S11), we obtained the mean level over the minor variants at 1.10% for the 2% mixtures and 1.13% for the 1% mixture in mixture II. Overall, 14 out of the 55 distinct positions show missing variants in at least one of the six samples in mixture I (Supplementary Table S10), whereas 28 out of the 43 expected variants show some missing variants over all six samples in mixtures II (Supplementary Table S11). For the 5% mixtures, all variants could be detected for mixture I as expected, while one variant was missing in mixture II (on position 13827 attributed to strand-bias in Mutserve2). For the 2% level for mixture I, only one variant was missing when applying the 1.5% threshold over both aligner/mappers, all other variants were present either one of the two mappers (minimap2 missing 3 or Ngmlr missing 2). For the 1% mixtures with their 0.7% cut-off, the rate of false negatives for the minor variants varied between 10/18 and 7/18 in mixture I and 10/23 and 11/23 in mixture II (for Ngmlr and Minimap2, respectively).

### 3.5 Accuracy of Common Variants

Finally, common variants were analyzed in more depth with Mutserve2 by varying the per-base quality filter (Supplementary Figures S9-S12). We expect the mutual homoplasmic variants from both samples of a mixture (previously confirmed via NGS) to be present with an AF of 100%. Thereby we can estimate and obtain an indication of the noise present in the data. We could note a significant difference between the variant levels over both mixtures when comparing the aligner/mapper Minimap2 and NGMLR. Overall experiments, Minimap2 yielded variant levels of 99.5% over the common variants, while NGMLR showed median variant levels at 99.1% (Wilcoxon, *p* = 2.2e^−16^). Both aligner/mapper had the lowest variant levels around 97% in the mixture I, while all common variants in mixture II were above 98% (Supplementary Figure S9).

## 4 Discussion

We aimed to investigate the applicability of nanopore long-read sequencing to detect low-frequency variants. To our knowledge, this is the first study that analyzes the performance of nanopore sequencing to detect variants down to an alternative AF of 1%. The precise determination of variants with low frequencies is of great importance across a wide range of fields, e.g. in the context of cancer research and the investigation of mosaic variants or the assessment of mitochondrial heteroplasmy (Watson et al., 2013; Stewart and Chinnery, 2015).

NGS is the most widely used technology for detecting low-frequency variants, and the accuracy of the method and different variant callers have been validated in the past (Kroigard et al., 2016; Fazzini et al., 2021; Pei et al., 2021). Likewise, TGS has become more accurate in recent years, and because of the longer read length, this technology holds a greater potential for structural variant calling. However, an in-depth validation of low-frequency variant calling from TGS data and evaluation of current limitations has not yet been performed.

The novelty of our study includes a successful benchmarking of nanopore long-read mitochondrial mixture models using a gold standard derived from NGS data. There are limitations to our gold standard, including sequencing artifacts (i.e. transversions), phantom mutations, and sequencing quality. These NGS artifacts could result in Type II errors in this study. To counteract systematic artifacts arising from NGS variants calling, three different callers were used and only variants detected with at least two tools were included in the gold standard.

Our results show that all steps of the data processing pipeline are of importance and can affect variant calling. With the new Guppy five super-accurate base-calling, we obtained overall higher base-calling q-scores compared to the older version 4. In addition, we detected fewer false-positive calls with a higher q-score (Supplementary Figure S7, 8), though hampered by lower coverage and fewer true-positive calls (Supplementary Figures S3, S7, S8). Thus, we used a minimum q-score of 20 for all tested variant callers to obtain sufficient base-calling quality and coverage, corresponding to a 1% per-base error rate. In addition to the q-score improvement in the past few years, a significant increase in nanopore sequencing accuracy is expected in the near future due to the advancement of the new R10.4 flow-cells and Kit 12 and Kit 14 Q20 + sequencing chemistry. Especially with regard to duplex base-calling (forward and reverse strand being sequenced and base-called), the accuracy of SNP and INDEL calling will increase.

We demonstrate the strengths and limitations of each mapper/aligner and variant caller pairs. For example, alignment with Ngmlr led to fewer false positives and higher F_1_ scores with Mutserve2 as the subsequent caller. In contrast, alignment with Minimap2 better reflected the expected variant levels of the common variants and led to lower variant levels among the false-positive calls. We also observed some “phantom-mutation” with higher levels for Ngmlr, compared to Minimap2, where we could identify homopolymorphic nucleotide stretches as the main cause. In addition to the aligner, the reference sequence could impact the variant calling. However, changing the reference to the major haplogroup would only be beneficial for detecting the major variants expected at 95%–99% depending on the mixture ratios from 5% down to 1%, which were all detected already. Thus, we would not expect an increase in the F1 score, as the detection of minor variants would not benefit from this approach.

Lastly and most significantly, the variant callers showed very different performances regarding the accurate detection of low-frequency variants. In our study, Mutserve2 showed the highest overall F_1_ scores. Using Freebayes, significantly more false positives were detected in comparison with the other two tested callers. In contrast, the lower F_1_ scores obtained with Nanopanel2 were due to the low number of true-positive variant calls, also including missing common and major variants. As Nanopanel2 is incapable of processing nanopore data from the super-accurate mode, this lower quality base-calling could be reflected in the lower F_1_ scores. As previously mentioned, the majority of false-positive variants calls were located within homopolymer stretches, which are known to be especially prone to systematic nanopore sequencing errors (Harris et al., 2019).

One mtDNA-specific phenomenon, which our data analysis workflow has not addressed, is transpositions of mitochondrial DNA into the nuclear DNA. NUMTs (nuclear mitochondrial DNA) have been challenging to delineate with short-read sequencing (Dayama et al., 2014), especially rare full-length or concatenated mtDNA copies in the nuclear genome (Wei et al., 2020). However, as most NUMTs are shorter than 500bp (Dayama et al., 2014), long-read sequencing can be beneficial to discriminate between NUMTs and real heteroplasmy. In our study, we performed long-range PCR and only included reads with a length >9kb, reducing the potential NUMTs contamination. Additionally, we did not find any potential NUMTs >1% after annotating with 1000 genomes project data (Dayama et al., 2014).

The validation of the low-frequency variant calling showed that all tested software had better performance with the mixture I, mainly due to the lower minor mixture component of the mixture II at the 2% level, which was, in reality, closer to a 1% level. This was a limitation from pipetting error at such small volumes. Thus, the F_1_ score of the 2% level of mixture I is more reflective for the future sequencing of real samples. One limiting factor of our study was that we could not compare all somatic variant callers. However, we do provide all data so that other researchers can compare different variant callers or methods in the post-processing. Another tool exclusively designed for nanopore sequencing data is Medaka, which creates a consensus sequence and calls variants (https://github.com/nanoporetech/medaka). However, using Medaka, we only detect major and common variants in our data set and no minor variants (low-frequency variants) (Supplementary Tables S12-S17), therefore, we did not focus further on this tool. Another limitation of our study is that we obtained uneven coverage between the two amplicons (fragments one and 2), which could bias the variant calling performance. Again, equimolar ratios of the PCR product were challenging to gauge and preferential barcode sequencing hampered the coverage across both amplicons in each sample. For short-read data, minimum coverage of 500X was recommended to detect variants with an AF>1% (Dierckxsens et al., 2020). For both PCR fragments, we have obtained a coverage >1000X, however, the base-calling accuracy of nanopore long-read data is considerably lower compared to Illumina short-read sequencing. Furthermore, it was noted that recombination because of PCR amplification could lead to chimeric reads that could potentially result in artificial mutations (Dierckxsens et al., 2020). Nevertheless, the high coverage obtained from the deep sequencing of the PCR amplicons was a significant benefit with regard to the lower read accuracy. On the other hand, due to the long-read sequencing, the coverage of individual fragments was even, spanning the complete PCR product. This is an essential advantage of TGS sequencing in comparison to NGS. As new variant callers will be developed eventually, our study provides a valuable TGS benchmarking data set for further validation.

## 5 Conclusion

In conclusion, our data show the feasibility of nanopore long-read low-frequency variant calling. However, the number of Type I and II errors increases between the 2% and 1% levels significantly. Here, the rather low per-base quality filtering (compared to current NGS data) of Q20 yields a higher number of false-positive variants, especially for variant callers designed for NGS data. Therefore, novel computational approaches are needed to disregard artifacts by taking advantage of almost complete haplotypes, as is the case with the short 16.6 kb mtDNA. Overall, we showed that all post-processing steps affect the final results, from base-calling and over aligning/mapping to variant calling tools. As further improvements in the data analysis pipeline arise and novel tools will become available for the mentioned steps, we herein provide a novel TGS benchmarking dataset.

## Data Availability

The datasets generated and analyzed for this study can be found in the Sequence Read Archive (SRA https://www.ncbi.nlm.nih.gov/sra) under the accession number: PRJNA814051 (SAMN26535982- SAMN26535987).
