# Supplementary material for: Benchmarking Low-Frequency Variant Calling With Long-Read Data on Mitochondrial DNA
Source: Front Genet. 2022 May 19;13:887644. doi: 10.3389/fgene.2022.887644 (PMC9161029; doi:10.3389/fgene.2022.887644)
Supplement: Supplementary file 1 [file DataSheet1.docx]

Supplementary Material

#
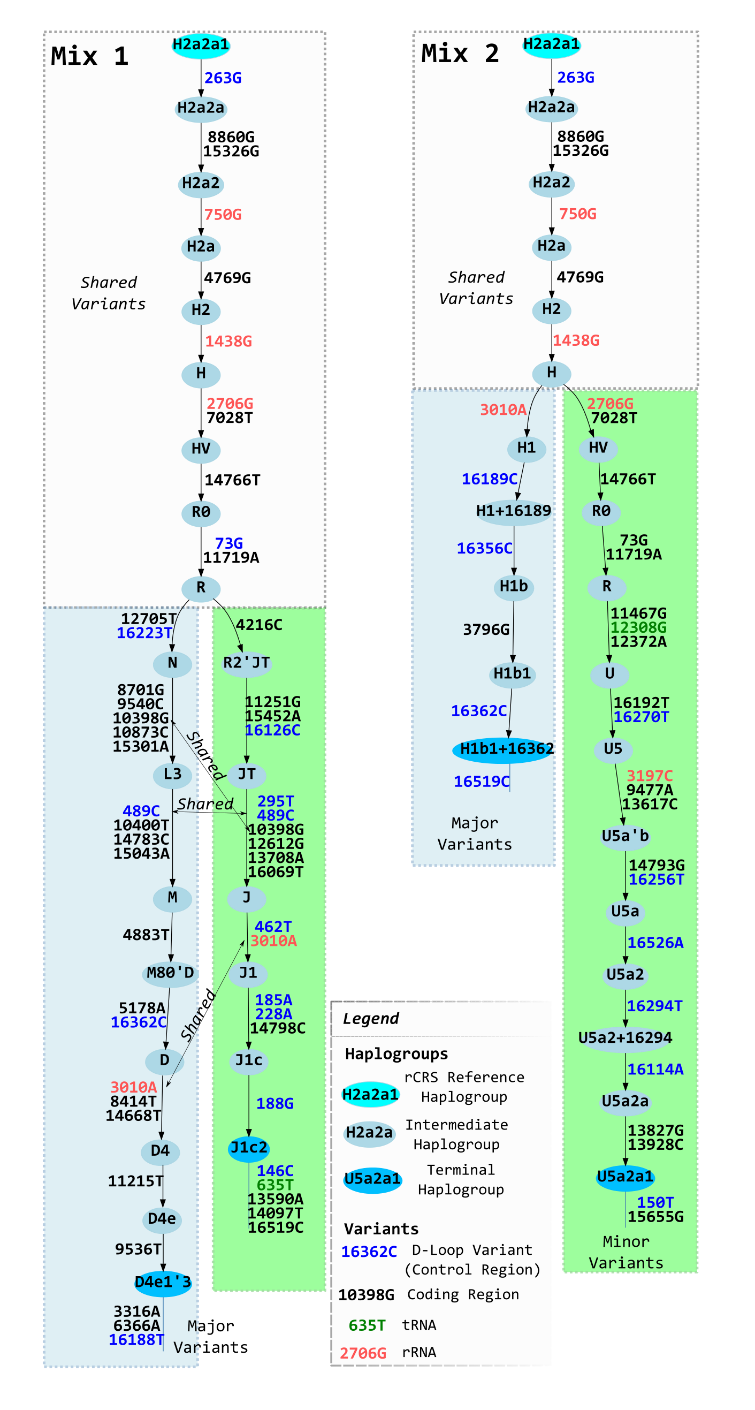
Supplementary Figures

**Supplementary Figure 1.** Overview of mixtures and variants expected, based on the gold-standard NGS samples. Mixture I has a sample with haplogroup D4e1'3 as major component (19 unshared major variants), and a sample with haplogroup J1c2 (18 unshared minor variants) and 14 shared variants, expected at the 100%. Mixture II consists of a sample from haplogroup H1b1+16362 (6 major variants) and U5a2a1 (22 minor variants) with 6 shared variants. The color denote the locus of a variant in either coding region, control region (CR), tRNA or rRNA.


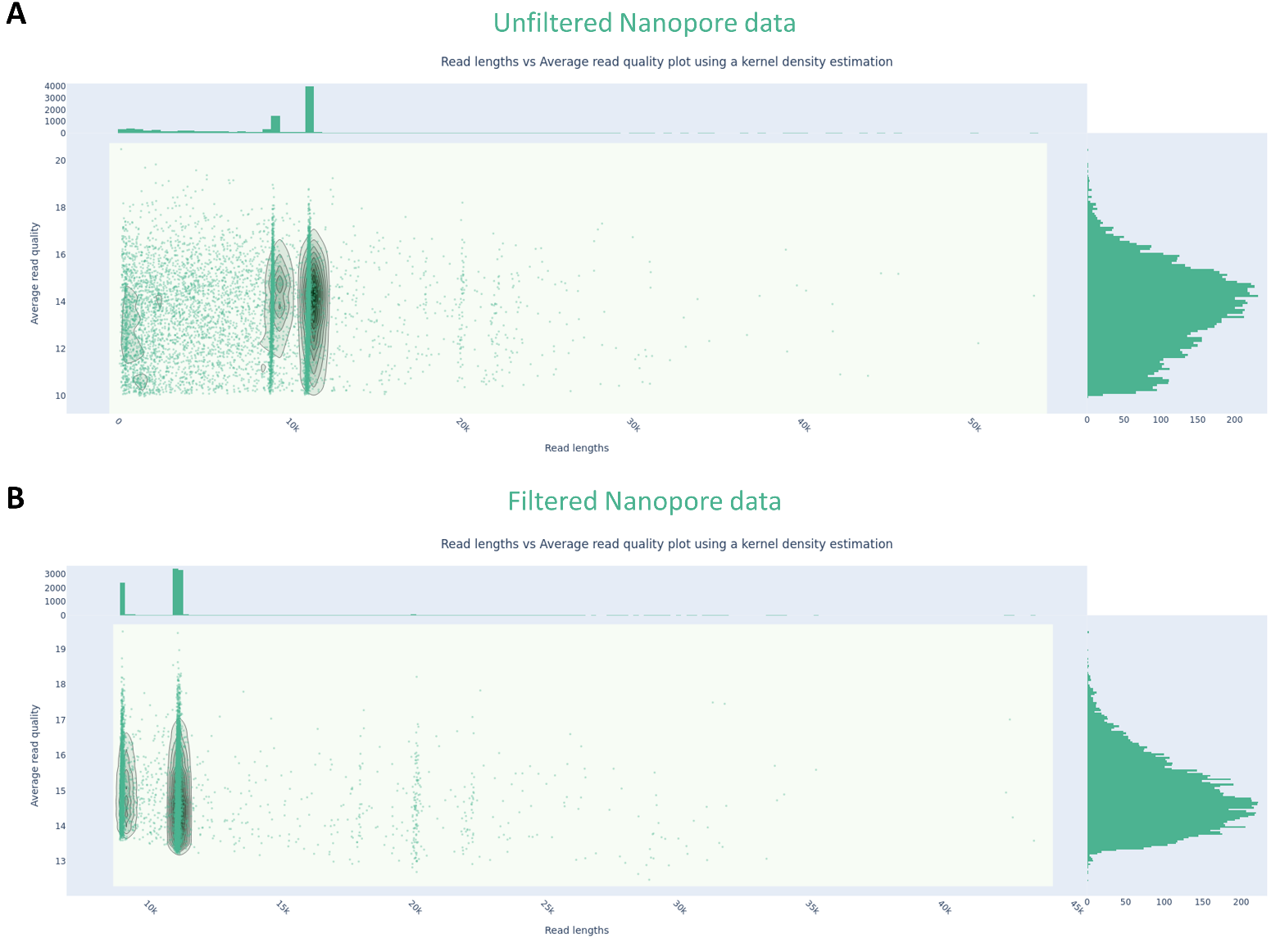
**Supplementary Figure 2. (A)** NanoPlot Report: Scatter plot of the average read quality and read length of all sequencing data. **(B)** NanoPlot Report: Scatter plot of the average read quality and read length of Nanopore data after quality filtering.


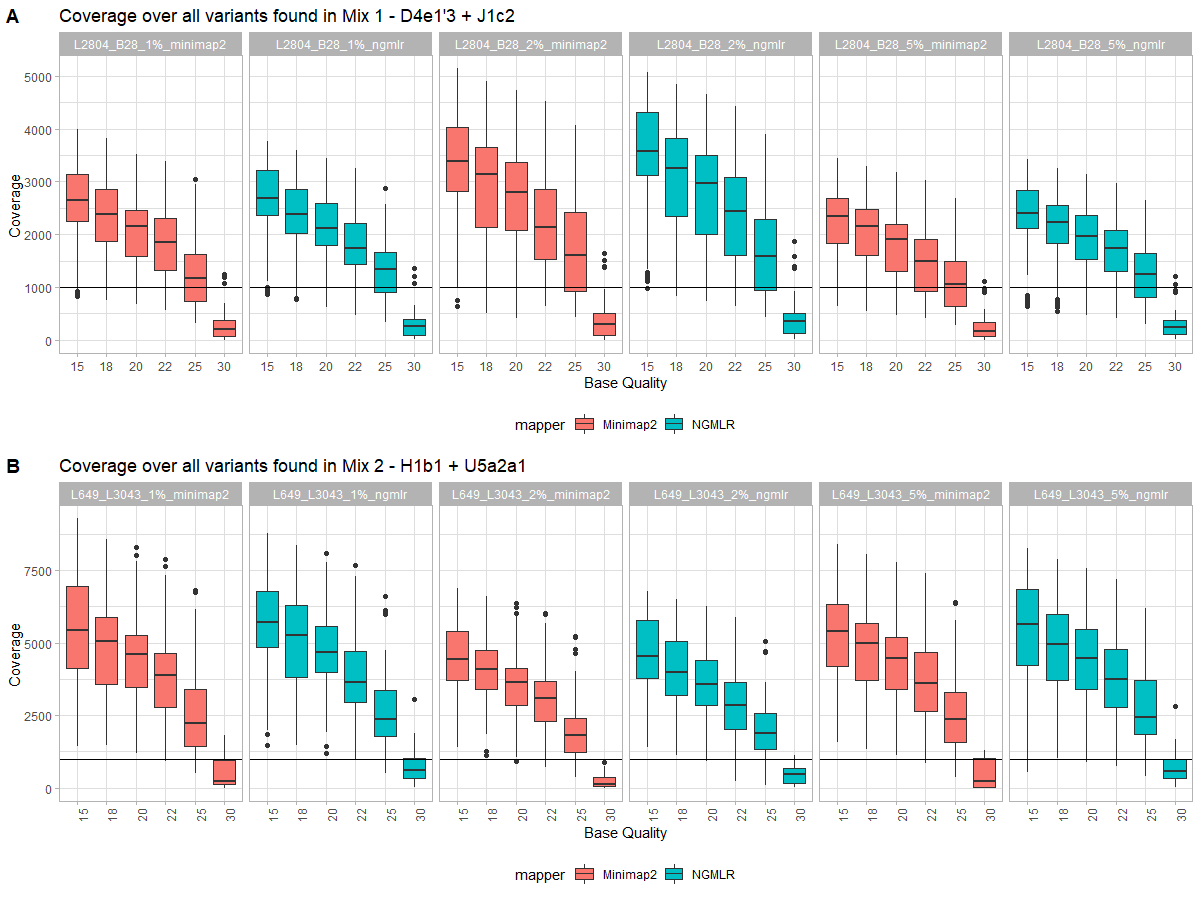
**Supplementary Figure 3.** Observed coverage in the six samples (three samples in mix 1 (mixture I), and three samples in mix 2 (mixture II)) over all variants called with Mutserve2, by adapting the parameter –baseQ, filtering between Phred Quality Score 10 (Error E= 10%), 15 (E= 0.0316), 18 (E= 0.0158), 20 (E=0.01), 22 (E= 0.0063), Q25 (E=0.00316) and 30 (E=0.001). Horizontal line denotes coverage 1000x as reference.


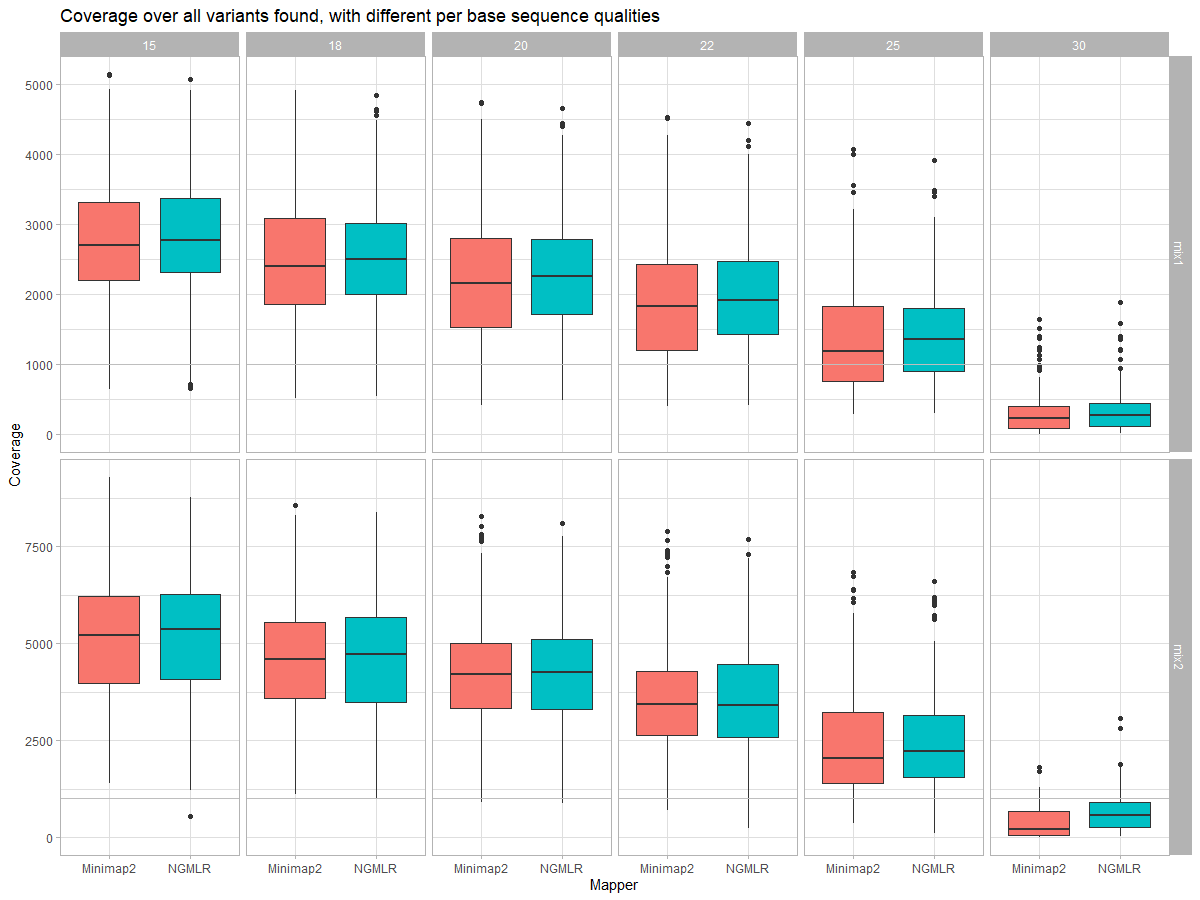
**Supplementary Figure 4.**. Detailed coverage over all variants in the different sample mixtures (red= Minimap2 1, blue=NGMLR). Phred Scores from 10 to 30 as present in S3. Horizontal line denotes coverage 1000x as reference.


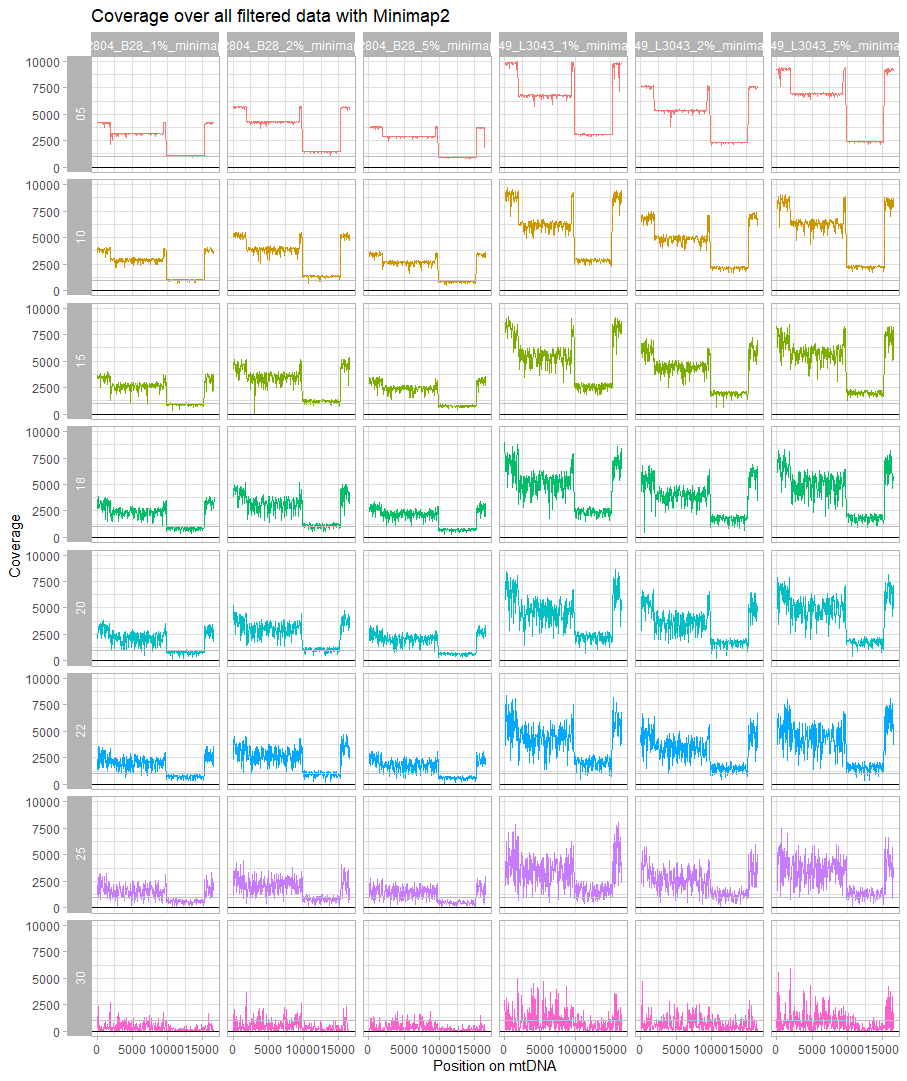
**Supplementary Figure 5.1**. Coverage plot over two mixture pairs at three different levels of 1%, 2% and 5% (all in one row), with different base quality filtering per row, up to Phred Score 30 (see Figure S3 for details of Phred-Score qualities and error rates). FASTQ files were aligned with Minimap2.


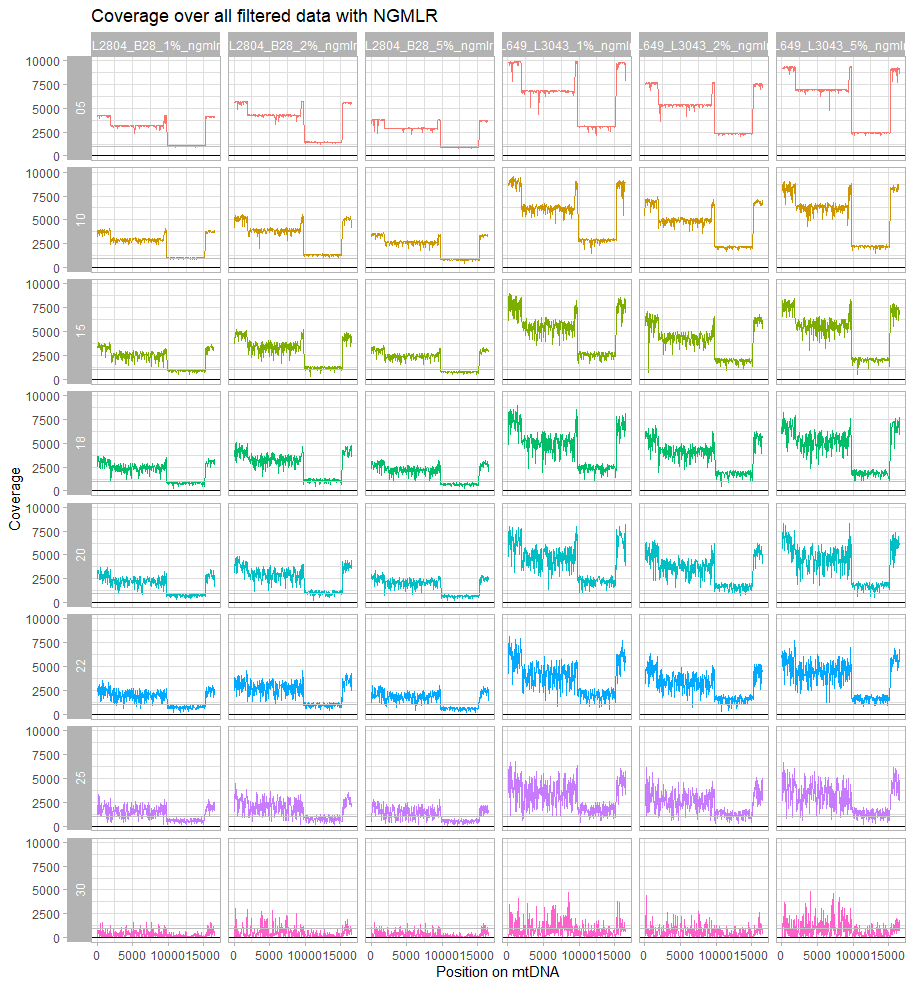
 **Supplementary Figure 5.2**. Coverage plot over two mixture pairs at three different levels of 1%, 2% and 5% (all in one row), with different base quality filtering per row, up to Phred Score 30 (see Figure S3 for details of Phred-Score qualities and error rates). FASTQ files were aligned with NGMLR.

**Supplementary Figure 6.** Relationship between the mixture percentage of the minor component and the F_1_ score **(A)** and the number of false-positives (n_FP_) **(B)** detected with Mutserve2. Mutserve2 parameters --alignQ 30, --mapQ 20 were used. Phred Scores of q=20 and heteroplasmy level of 0.005 was used. r = Spearman’s rank correlation coefficient, p = Spearman’s exploratory p-value.


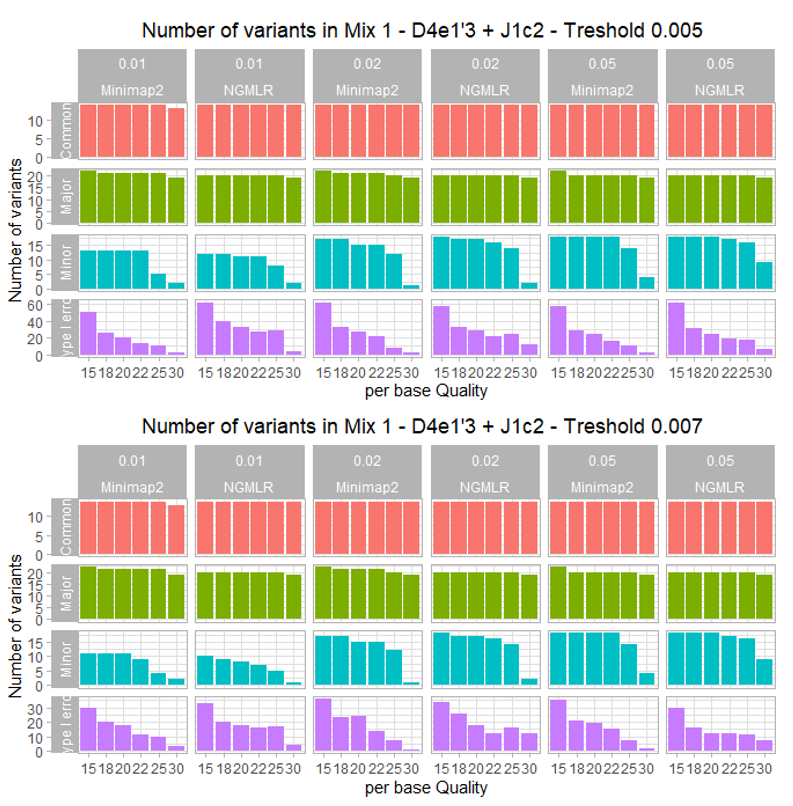


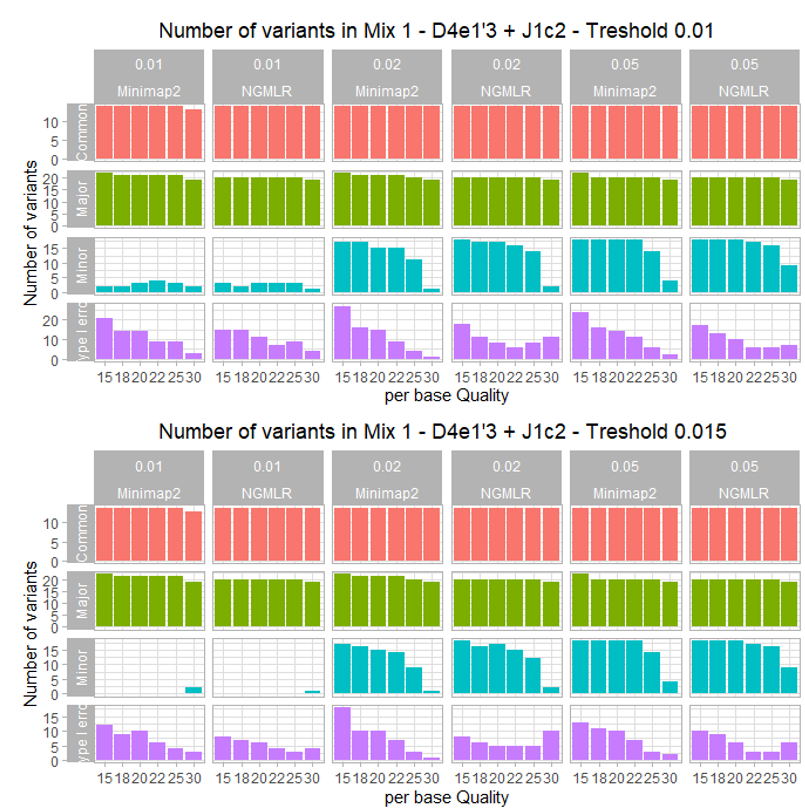
**Supplementary Figure 7.1-7.4.** Number of variants detected per variant type in mixture 1. Types comprise Common (shared variants), major (variants found in D4e1’3), minor (variants expected from J1c2) and false positives type I error in the gold-standard. Mutserve2 parameters - -alignQ 30, --mapQ 20. Phred Scores from 15 to 30 as present in S3 and minimum heteroplasmy level from 0.005 to 0.015 was used.


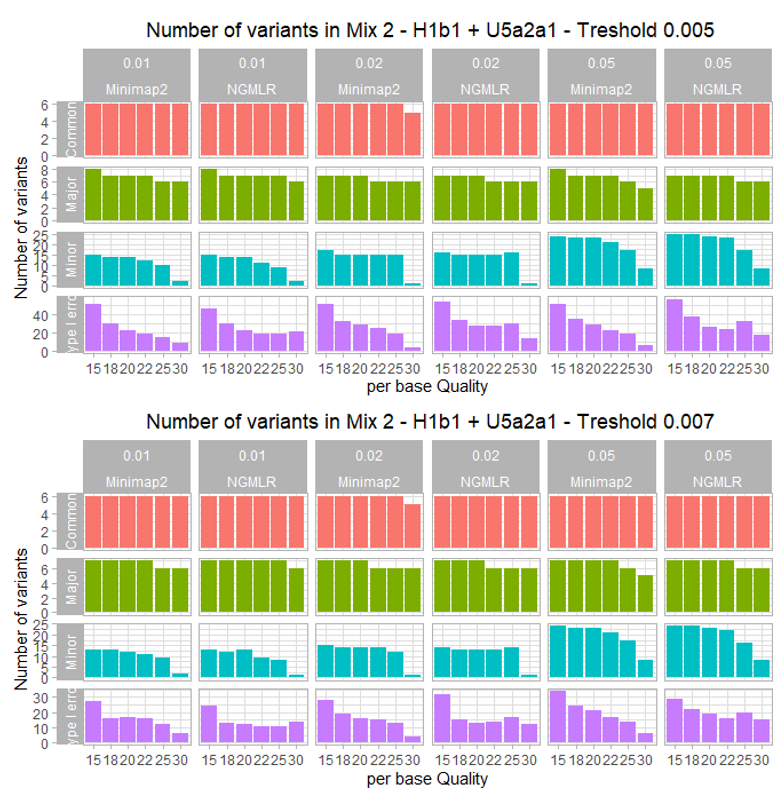


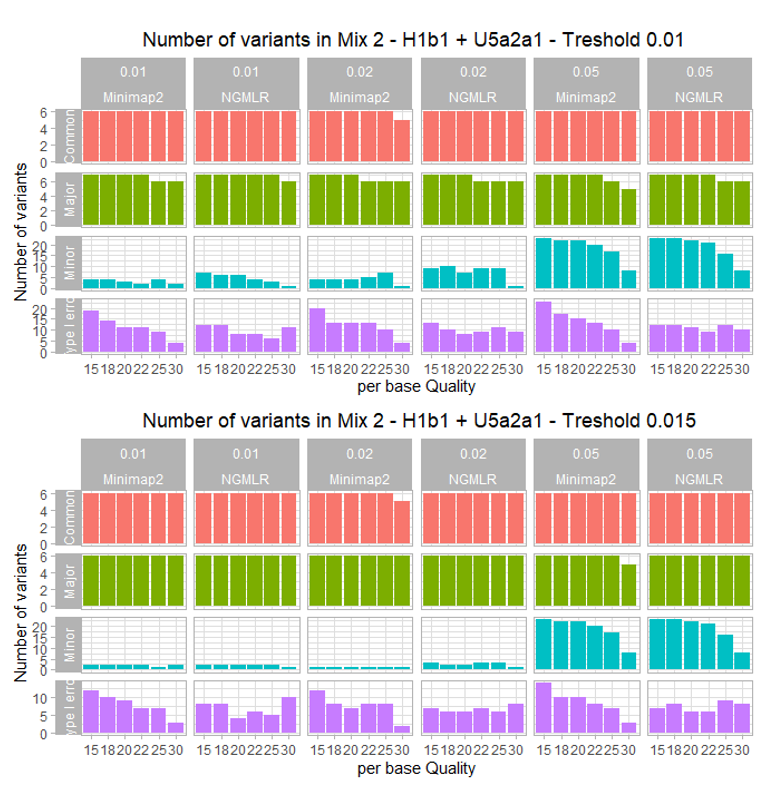
**Supplementary Figure 8.1-8.4.** Number of variants detected per variant type in mixture 2. Types comprise Common (shared variants), major (variants found in D4e1’3), minor (variants expected from J1c2) and false positives type I error in the gold-standard. Mutserve2 parameters - -alignQ 30. Phred Scores from 10 to 30 as present in S3 and minimum heteroplasmy level from 0.005 to 0.015 was used.


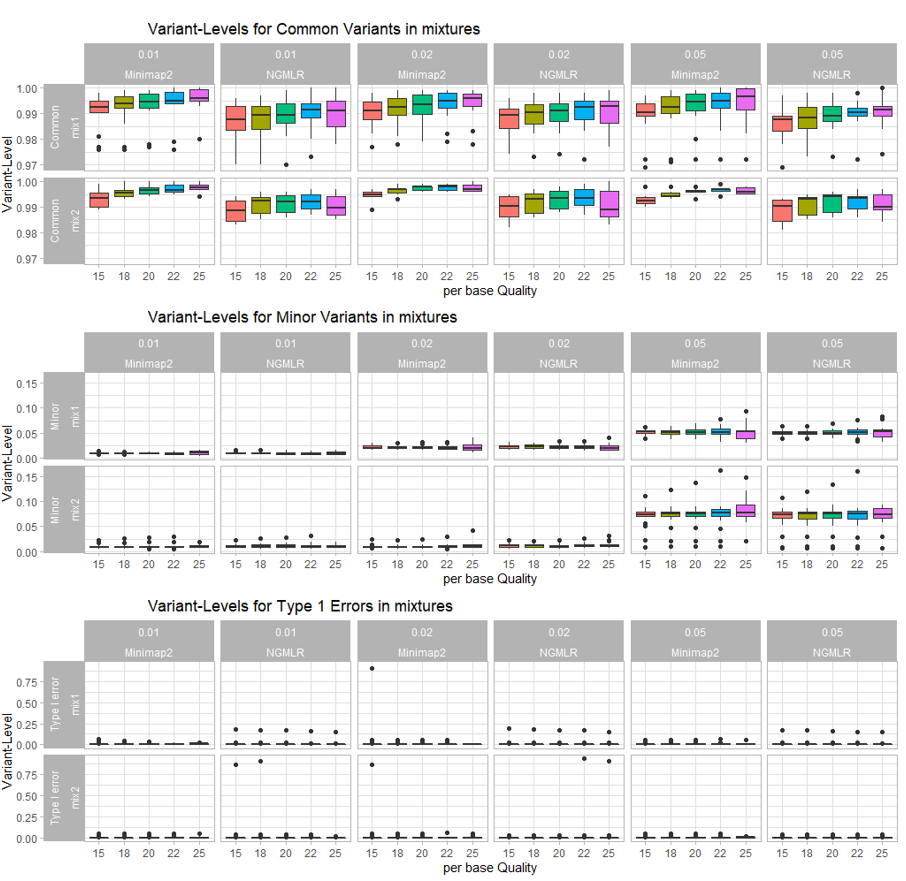


**Supplementary Figure 9.1-9.3**. Variant-Levels observed in mixture1 and mixture 2. For all samples the same variant-level threshold was applied in Mutserve2. Variant types as presented in previous figure S7. Phred Scores from 10 to 30 as present in S3.


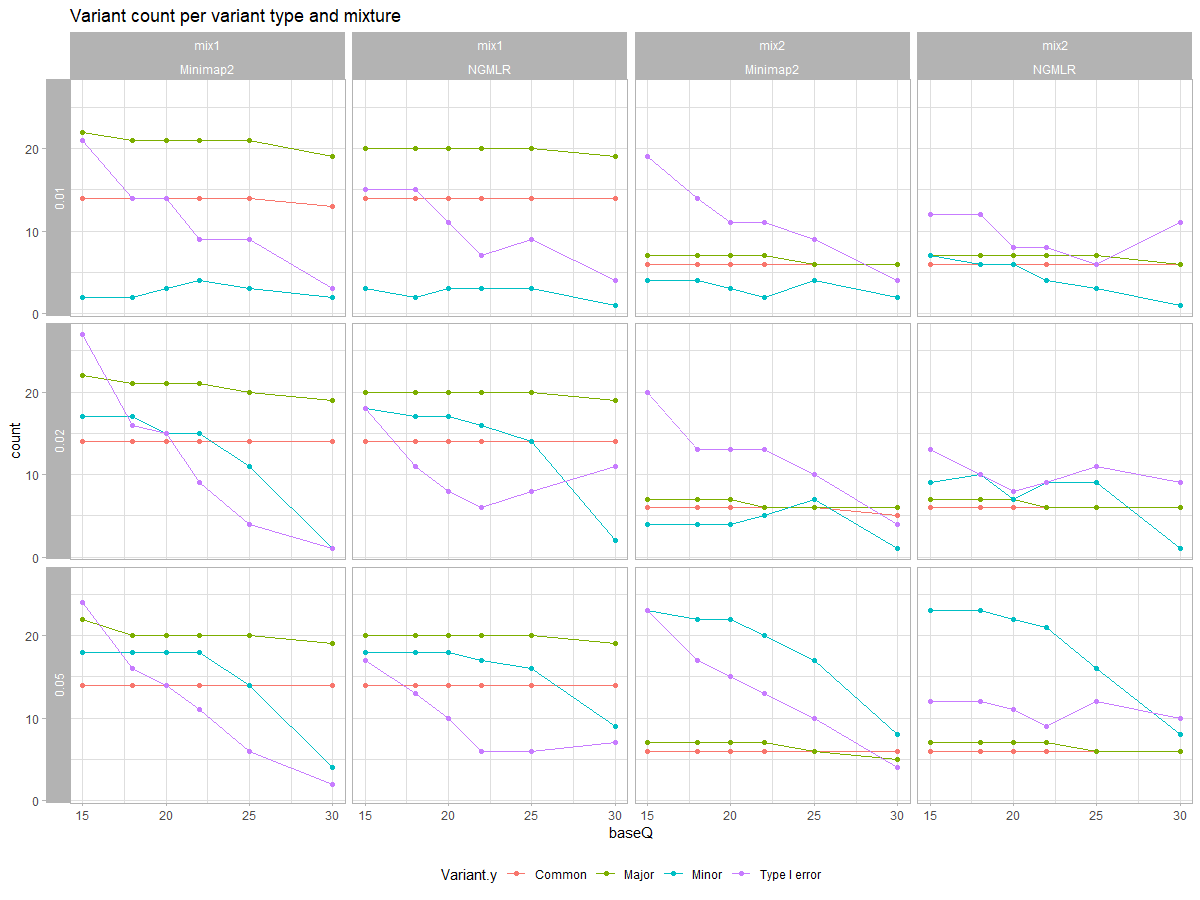
**Supplementary Figure 10.** Variant count per variant type stratified for mixture and aligner. Phred Scores from 10 to 30 as present in S3.


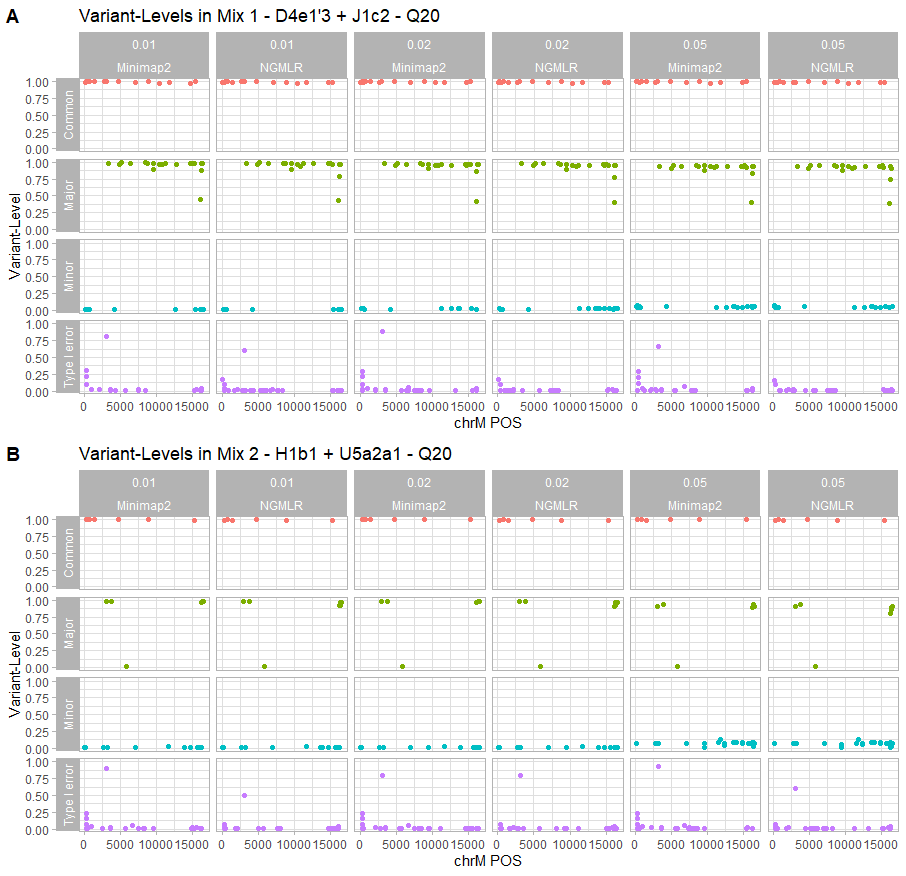
 **Supplementary Figure 11.** Variant-Levels observed in mixture1 (A) and mixture 2 (B) at the per-base quality threshold 20. For all samples the same variant-level threshold was applied in Mutserve2 Variant types as presented in previous figure S7 and the minimum heteroplasmy level of 0.005 was used.


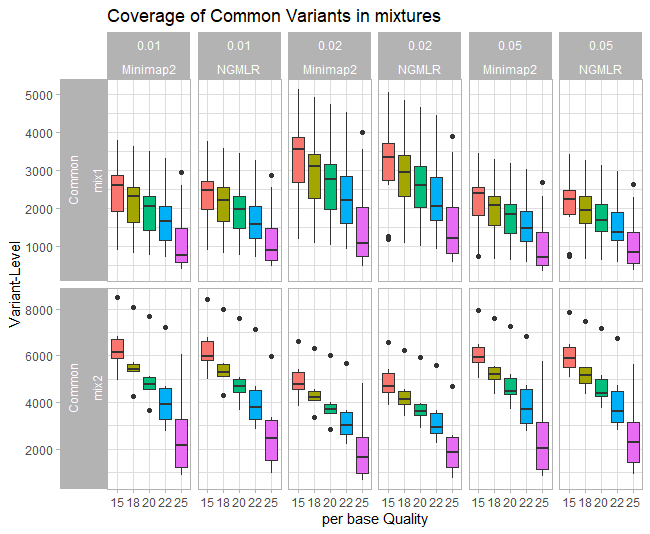
**Supplementary Figure 12.** Observed coverage in the six samples (three samples in mix 1, and three samples in mix 2) over all common variants called with Mutserve2. Phred Scores from 10 to 30 as present in S3.

# Supplementary Text 1

**Preparation of Goldstandard from Illumina short read data – Variant calling steps:**

#freebayes 1.3.1

for i in *.bam; do

freebayes-v1.3.1 -f rcrs.fasta -b $i --min-mapping-quality 30 --min-base-quality 20 --min-alternate-fraction 0.01 --min-alternate-count 10 --ploidy 1 > "$i".freebayes.vcf

bcftools query -f '%POS\t%REF\t%ALT\t[%GT]\t[%AD] \n' "$i".freebayes.vcf > "$i".freebayes.txt

done

#GATK Mutect2 Version:4.1.8.1-2-gf53087d-SNAPSHOT

for i in *.bam; do

gatk Mutect2 -R rcrs.fasta -L rCRS --min-base-quality-score 20 --minimum-allele-fraction 0.01 --mitochondria-mode -I $i -O "$i".gatk.vcf;

bcftools query -f '%POS\t[%GT]\t[%AD]\t[%AF] \n' "$i".gatk.vcf > "$i".gatk.txt

done

#mutserve v2.0.0-rc8

java -jar mutserve.jar call --reference chrM.fasta *.bam --threads 8 --out mutserve2_out.vcf

For each variant caller we obtain a Tab-delimted text file. For each sample we merge the three different variant caller results, and consider a variant if at least 2 of 3 variant callers confirm it. See Supplementary Table 2 for NGS gold standard

**The Nanopore sequencing data of all samples were processed in the same following way.**

1) The FASTQ files that passed the Guppy base-calling were filtered with Filtlong (v0.2.0) to only include the best 50% of the reads, based on Phred quality scores (q-score), and reads >9kb. Subsequently the reads were trimmed with NanoFilt (v2.5.0).

filtlong --min_length 9000 --keep_percent 50 $i.fastq > $i.filt.fastq

cat $i.filt.fastq | $PathToNanoFilt/NanoFilt -q 12 --headcrop 75 --tailcrop 20 > $i.filt.trimed.fastq

2) Next, the reads were aligned with Minimap2 (v2.17) and Ngmlr (v0.2.7) to the mitochondrial genome (rCRS).

minimap2 -a -x map-ont $FASTA $i.filt.trimed.fastq > $i.filt.mm2.sam

ngmlr -t 4 -r $FASTA -q $i.filt.trimed.fastq -o $i.filt.ngmlr.sam -x ont

3) The alignments from Minimap2 or Ngmlr were converted to BAM format and subsequently sorted and indexed with Samtools (v1.3.1).

samtools view -S -$i.filt.mm2.sam/$i.filt.ngmlr.sam > $i.filt.mm2.bam/$i.filt.ngmlr.bam

samtools sort $i.filt.mm2.bam/$i.filt.ngmlr.bam -o $i.filt.mm2.sort.bam/$i.filt.ngmlr.sort.bam

samtools index $i.filt.mm2.sort.bam/$i.filt.ngmlr.sort.bam

4) The alignment in the BAM format obtained from Ngmlr or Minimap2 was used to call variants with Mutserve2 (v2.0.0) and Freebayes (v1.3.4)

4.1) Mutserve2:

mutserve call --reference $FASTA --output $RESULTS/Mixture_final.txt --threads 16 \ $PathToBamMM2andNGMLR/ --level 0.005

mutserve call --reference $FASTA --output $RESULTS/Mixture_final.txt --threads 16 \ $PathToBamMM2andNGMLR/ --level 0.005 --write-raw --level 0.005

mutserve annotate --input $RESULTS/Mixture_final.txt --annotation $ANNOTATIONFILE \

--output $RESULTS/Mixture_final.annotated.txt

4.2) Freebayes

freebayes -f $FASTA -b $i.filt.mm2.sort.bam/$i.filt.ngmlr.sort.bam \

--throw-away-indel-obs --use-best-n-alleles 4 -C 5 \

--throw-away-mnps-obs --haplotype-length 0 --pooled-continuous -F 0.005 -m 20 -q 20 \

> "$i".mm2.freebayes_final.vcf/"$i".ngmlr.freebayes_final.vcf

5) To call variants with Nanopanel2 (v1.01), the Nanopore FAST5 files had to be rebase-called with Guppy v4.5.4 in high accuracy mode and then the FASTQ files were processed the same way as described above. On the basis of the filtered and trimmed FASTQ files, the FAST5 files were sub-setted as well with ont_fast5_api. The resulting filtered FASTQ and FAST5 files were used as the input to call variants with Nanopanel2. The alignment with either Minimap2 or Ngmlr is integrated into the processing of Nanopanel2 and the aligner can be defined in the config file

fast5_subset --input $PathToUnfiltFast5/ --save_path $PathToFiltFast5/ \

--read_id_list $PathToFiltFastqIDs/$i.filt.trimed.ID.txt --batch_size 4000 --recursive

cd $RESULTS

singularity run $PathToNanopanel2/nanopanel2_1.01.sif call \

--conf $PathToJsonFile/Mitoseq_config.json --out .

# Supplementary Tables

**Supplementary Table 1.** Overview of participants selected for the study.

| **ID** | **B-28** | **L-2804** | **L-3034** | **L-649** |
| --- | --- | --- | --- | --- |
| **Age** | 37 | 75 | 53 | 48 |
| **Population** | Bolzano | German | German | German |
| **Haplogroup** | D4e1’3 | J1c2 | H1b1 + 16362 | U5a2a1 |

**Supplementary Table 3. Mitochondrial DNA Long-range PCR mix for one sample.**

| **Reagent** | **1x [µl]** |
| --- | --- |
| Takara GXL Buffer | 10.0 |
| dNTPs | 4.0 |
| 10µM Primer F | 1.0 |
| 10µM Primer R | 1.0 |
| Takara GXL Polymerase | 1.0 |
| DNA (50-100ng) | X |
| Nucelase free water | 50-X |
| ∑ | 50.0 |

**Supplementary Table 4. Long-range PCR cycling conditions for amplifying the two overlapping mitochondrial amplicons.**

| **Temperature and duration** | **Process and cycles** |
| --- | --- |
| 94°C for 1:00 | Initial denaturation |
| 94°C for 0:15 | 14 cycles |
| 60°C for 0:30 |  |
| 68°C for 5:00 |  |
| 94°C for 0:15 | 27 cycles |
| 57°C for 0:30 |  |
| 68°C for 9:00, increase 0:10 per cycle |  |
| 72°C for 5:00 | Final extension |
| 4°C for ever | ---- |

**Supplementary Table 5. Preparation of the mixtures of the PCR products at predefined 1%, 2% and 5% ratio.**

| **Mixture I** | | | |
| --- | --- | --- | --- |
|  | | **Major component: D4e1’3** | **Minor component: J1c2** |
| **Volume of PCR product [µl]** | **1%** | 48.5 | 0.5 |
|  | **2%** | 48.0 | 1.0 |
|  | **5%** | 46.6 | 2.5 |
| **Mixture II** | | | |
|  | | **Major component: H1b1+16362** | **Minor component: U5a2a1** |
| **Volume of PCR product [µl]** | **1%** | 48.5 | 0.5 |
|  | **2%** | 48.0 | 1.0 |
|  | **5%** | 46.6 | 2.5 |
